# Editorial Note: How institutional quality, and energy production sources, affect the environmental sustainability of bri countries: A comparison of different income groups

**DOI:** 10.1371/journal.pone.0337372

**Published:** 2025-11-25

**Authors:** The PLOS One Editors

The *PLOS One* Editors issue this Editorial Note to inform readers that we have concerns about aspects of the article’s peer review. After reviewing this matter PLOS concluded that the article’s publication is supported based on expert input that was not affected by the concerns. We regret that the issues were not addressed prior to the article’s publication.
